# O053: What surgical site infection rates in colorectal surgery should be considered for benchmarking standards?

**DOI:** 10.1186/2047-2994-2-S1-O53

**Published:** 2013-06-20

**Authors:** E Shaw, JM Badia, M Piriz, R Escofet, E Limón, F Gudiol, M Pujol

**Affiliations:** 1 Infectious Diseases, Hospital Universitari de Bellvitge, Hospitalet Llobregat, Spain; 2 General Surgery, Hospital de Granollers, Granollers, Spain; 3 Infection Control, Hospital Parc Tauli, Sabadell, Spain; 4 Infection Control, Hospital Universitari de Bellvitge, Spain; 5 Vincat Program, Departament de Salut, Hospitalet Llobregat, Spain

## Introduction

Surgical site infection (SSI) after colorectal procedures represents a measurable quality indicator of a health care system. There is interest in comparing SSI rates between different hospitals and countries, however variability of the data regarding to incidence of SSI makes this comparison controversial. For the purposes of evaluation, data must be standardized and include reliable post-discharge surveillance (PDS).

## Objectives

To determine rates of SSI after elective colorectal procedures among hospitals of the VINCat Program.

## Methods

VINCat is a nosocomial infection surveillance program in Catalonia, Spain. Between 2007 and 2012, 59 hospitals joined the program. The participating hospitals performed active, prospective, standardized surveillance of elective colorectal resection. PDS was implemented by a multimodal approach and was mandatory within the first 30 days after surgery. Since 2011 colon and rectal procedures were also analysed separately.

## Results

During the study period, 17,779 elective colorectal procedures were included. Mean age was 69y (SD:12y) and 40% were female. SSI was diagnosed in 3,485 (20.3%) patients. Among them, 782 (22.4%) were diagnosed during PDS. Median time from surgery to infection was seven days (IQR 5-9) for in-hospital SSI and 14 days (IQR 10-19) for PDS-SSI. Surgical infections due to colon procedures were only slightly lower (18.8%) than those due to rectal surgery (22.3%). Both, overall SSI rates and organ/space SSI rates did not change significantly over the study period and were respectively: 2007 (20.8%/5.3%), 2008 (19.2%/6.9%), 2009 (21%/9%), 2010 (21%/8.5%), 2011 (20.7%/9.3%) and 2012 (19%/8.9%).

## Conclusion

SSI rates in elective colorectal procedures at VINCat hospitals remained stable over the study period and were higher than those reported by other national programs. There is a need to clarify what surgical site infection rates in colorectal surgery should be considered for benchmarking standards.

## Disclosure of interest

None declared.

